# Targeted-pig trial on safety and immunogenicity of serum-derived extracellular vesicles enriched fractions obtained from Porcine Respiratory and Reproductive virus infections

**DOI:** 10.1038/s41598-018-36141-5

**Published:** 2018-11-30

**Authors:** Sergio Montaner-Tarbes, Elena Novell, Vicens Tarancón, Francesc E. Borrás, Maria Montoya, Lorenzo Fraile, Hernando A. del Portillo

**Affiliations:** 1 Innovex Therapeutics S.L, Badalona, Spain; 2 Departamento de Ciència Animal, ETSEA, Avenida Alcalde Rovira Roure, 191, Universidad de Lleida, Lleida, Spain; 3 Grup de Sanejament Porci, Lleida, Spain; 4 Germans Trias i Pujol Health Science Research Institute (IGTP), Can Ruti Campus, 08916 Badalona, Spain; 5 ISGlobal, Hospital Clínic - Universitat de Barcelona, C/ Roselló 153, 08036 Barcelona, Spain; 6 Institució Catalana de Recerca i Estudis Avançats (ICREA), Passeig Luis Companys 23, 08010 Barcelona, Spain

## Abstract

The Porcine Reproductive and Respiratory Syndrome Virus (PRRSV) is the etiological agent of one of the most important swine diseases with a significant economic burden worldwide. Unfortunately, available vaccines are partially effective highlighting the need of novel approaches. Previously, antigenic viral proteins were described in serum-derived extracellular vesicles (EVs) from pigs previously infected with PRRSV. Here, a targeted-pig trial was designed to determine the safety and immunogenicity of such extracellular vesicles enriched fractions. Our results showed that immunizations with EV-enriched fractions from convalescence animals in combination with montanide is safe and free of virus as immunizations with up-to two milligrams of EV-enriched fractions did not induce clinical symptoms, adverse effects and detectable viral replication. In addition, this vaccine formulation was able to elicit specific humoral IgG immune response in vaccinated animals, albeit variably. Noticeably, sera from vaccinated animals was diagnosed negative when tested for PRRSV using a commercial ELISA test; thus, indicating that this new approach differentiates vaccinated from infected animals. Lastly, after priming animals with EV-enriched fractions from sera of convalescence animals and boosting them with synthetic viral peptides identified by mass spectrometry, a distinctive high and specific IFN-γ response was elicited. Altogether, our data strongly suggest the use of serum EV-enriched fractions as a novel vaccine strategy against PRRSV.

## Introduction

Recent estimates calculate that the world human population will reach near 9,6 billion people by 2050 and the United Nations Food and Agriculture Organization (FAO) estimates that in order to feed this population the overall food production will need to increase by 70%^1^. Undoubtedly, veterinary vaccines that preserve animal health and improve production will play an essential role in reaching this goal. Moreover, they will reduce the use of antibiotics as they are escalating into a global health crisis. To reach this goal, novel vaccination approaches are desperately required as classical and live-attenuated vaccines are far from being totally safe and efficacious in animal diseases^2^.

The Porcine Reproductive and Respiratory Syndrome Virus (PRRSV) causes one of the most important diseases of veterinary medicine^3^. Recent economical estimates in the United States, where the disease is highly prevalent, indicate more than 600 million $ loses each year^4^. Current vaccines against PRRSV use modified live or attenuated virus, small peptides, vectored vaccines, inactivated virus and subunit vaccines^5^. Nevertheless, available vaccines have serious limitations such as little protective immunity, possible reversion to virulence, inability to induce long lasting and heterologous protection among European and American genotypes, and high antigenic and genetic differences of strains. All together, these limitations indicate that new alternatives to conventional vaccines are needed trying to control and eventually eradicating PRRSV.

Extracellular vesicles (EVs) are gaining increase scientific attention as novel vaccines against infectious diseases, including animal diseases of veterinary importance^6–9^. Our group previously determined that EVs obtained from serum of animals that had overcome a PRRSV infection were free of virus as detected by a commercial and sensitive qRT-PCR test and contained antigenic viral-specific cargo^7^. Here, we report a targeted-pig safety and immunogenicity trial by immunizing pigs with serum-derived EV-enriched fractions from convalescence animals. Our results revealed that these EV-enriched fractions are safe, free of virus and contained immunogenic viral peptides capable of eliciting specific humoral and cellular immune responses. Moreover, this approach seems capable of differentiating vaccinated from infected animals (DIVA).

## Material and Methods

### Ethical statement for experimental procedures

All studies were approved by the ethical committee of Universitat de Lleida and the Departament d’Agricultura, Ramaderia, Pesca, Alimentació I Medi rural (Section of Biodiversity and hunting) under licence DAAM 7700. All procedures and experiments in this research were performed in accordance with guidelines and regulations of University of Lleida and the Department of Animal Sciences of this University under the supervision of a veterinary.

### Serum samples

Serum was obtained from five individual large white-Landrace pigs of 80–100 kg of body weight that had suffered a PRRSV natural infection. Animals were anesthetized and approximately 1 L of peripheral blood from each animal was collected by venous puncture. Afterwards, animals were humanely euthanized according to procedures approved by the University of Lleida. Blood was collected into 50 mL Falcon tubes to facilitate separation of sera and minimize haemolysis. Viral as well as serological status of animals against PRRSV antigens were analysed, by an independent laboratory specialized in diagnosis of porcine diseases (Grup de Sanejament Porci (http://www.gsplleida.net/cat) using RT-PCR TaqMan® NA/EU PRRSV Reagents (Applied Biosystems) and IDEXX PRRS X3 Antibody Test (IDEXX) following their own standard operational procedures. All those animals were negative by PCR and positive for antibodies against PRRSV in serum.

### EVs enrichment and isolation

Enrichment of serum-derived EVs was obtained through ultracentrifugation^10^ followed by Size Exclusion Chromatography (SEC) using our own standard methodologies^11^. Sera samples were centrifuged for 2 hours at 100.000 × g at 4 °C and resuspended in ten mL of PBS (final volume) before loading into PuriFlash Dry Load Columns 80 G (Interchim) filled with 100 mL of sepharose CL-2B (separation matrix) and five mL fractions collected for further analysis. Nanoparticle tracking analysis (NTA), flow cytometry (FACS/culture supernatant monoclonal antibodies against EVs tetraspanins CD9, CD63, CD81 and CD5L from Abcam ab45408) and proteomic analyses (liquid chromatography/mass spectrometry: LC-MS/MS) were carried out as previously described^7^. Protein concentration was determined by bicinchoninic acid assay protein assay (BCA Pierce protein quantification assay – Thermo Scientific)^12^ and used as vaccine dose unit. All those tests are recommended as standard proxy for characterization of extracellular vesicles and exosomes^13^_._

### Mass spectrometry

Liquid chromatography (nanoLCULTRA-EKSIGENT) followed by mass spectrometry (LTQ Orbitrap Velos - Thermo Fisher) was used to identify viral proteins associated to EVs enriched fractions as previously described^7^. Briefly, protein identification was done using RAW data from five different animals from which EVs enriched fractions were analysed by LC-MS/MS and Maxquant software v1.5 excluding those hits identified by reverse database, marked as contaminant and identified by modifications. Only those proteins with 2 or more unique peptides and 1% FDR were used for further analyses.

### Synthetic peptides

After LC-MS/MS identification of PRRSV proteins associated with serum-derived EVs enriched fractions (Supplementary Table T1), two different algorithms were used to examine matching regions between our identified peptides (LC-MS/MS) and possible B-cell epitopes or antigenic regions within the protein^14,15^. Peptides corresponded to the Envelope glycoprotein (ENV), Nucleocapsid (N) and polyprotein 1a (pp1a) viral proteins. Matching regions were detected and then evaluated for suitable Swine leukocyte antigen binding (SLA) by NetMHCpan 4.0^16,17^. Those with higher score were used to synthetized 35aa peptides (Department of Experimental and Health Sciences – Peptide Synthesis Facility of Universitat Pompeu Fabra at Centre for Genomic Regulation (Barcelona – Spain) according to their own standard operation procedures. Characterization of each synthetic peptide was carried out using an A-HPLC with a Column Luna C18 (4.6 × 50 mm, 3 um; Phenomenex), a Gradient: Linear B (0.036% TFA in MeCN) into A (0.045% TFA in H2O) over 15 minutes with a flow rate of 1 mL/min and detection at 220 nm. All peptides were 90% pure and were resuspended in ultrapure H_2_O (MiliQ water), aliquoted and stored in −20 °C until use.

### Safety pig targeted trial

Fifteen PRRSV negative pigs were divided into groups with food and water *ad libitum* in an experimental farm (Centre d’Estudis Porcins, Torrelameu, Spain) under veterinarian supervision. EV-enriched fractions from animals that had overcome the disease were combined with Montanide ISA 206 VG (SAFIC-ALCAN, batch. T83571 - gently provided by SEPPIC) in a 1:1 ratio using three different EV-enriched fraction protein concentrations (0.3, 0.5 and 1 mg) and injected intra-muscularly (IM) in one side of the neck (Table 1). Each animal received 2 mL of the formulation in two different time points. Serum samples were drawn at 0, 21 and 51 days post initial vaccination (dpv). Collection of samples, maintenance and culling of pigs were performed as approved by the animal use and care committee protocol of the Universitat de Lleida and the Departament d’Agricultura, Ramaderia, Pesca, Alimentació I Medi rural (Section of Biodiversity and hunting) under licence DAAM 7700.

### Immunogenicity pig-targeted trial and challenge

**Figure 1:**
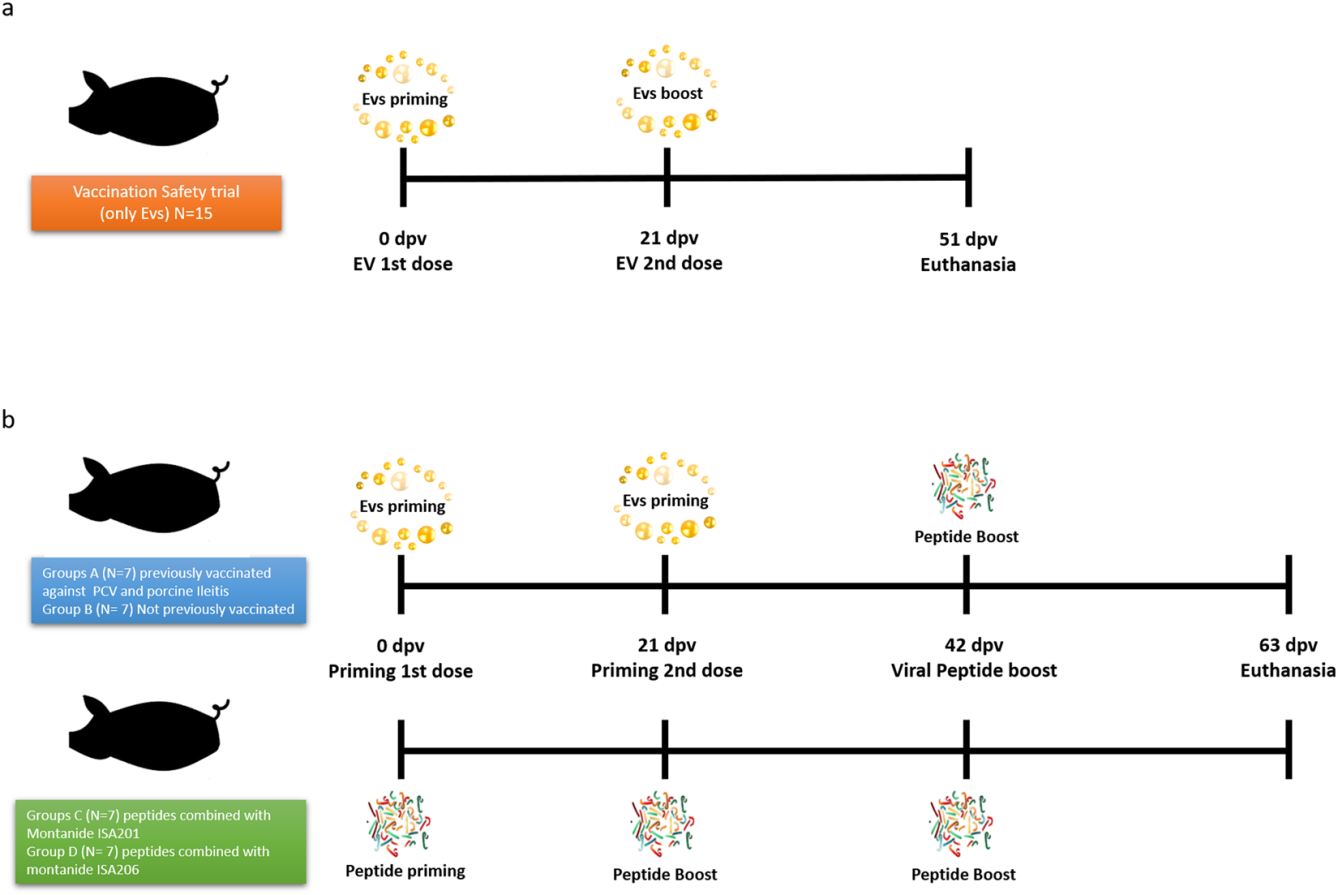
Scheme of safety and immunogenicity targeted-pig trial. Timeline of immunizations was the same for both strategies. (**a**) For safety trial, animals receive two doses of EVs injected intramuscularly (Day 0 and 21) and euthanised at day 51. (**b**) For immunogenicity trial, animals received either two doses of EV-enriched fractions or synthetic viral peptides (day 0 and 21) and all then boosted with synthetic viral peptides (day 42). All animals were euthanised at day 63. Serum samples were collected in all timepoints.

A heterologous prime-boost vaccination approach^18^ was performed using serum derived EV-enriched fractions as a prime antigen (2 doses) and a boost with the viral synthetic peptides identified by LC-MS/MS. First, thirty-three PRRSV negative pigs were divided into groups with food and water *ad libitum* in an experimental farm (Centre d’Estudis Porcins, Torrelameu, Spain) under veterinarian supervision. To test immunogenicity, 14 animals received one immunization and a boost containing 2 mL with 1 mg of EV-enriched fractions in different timepoints (Group A: completely naïve animals/Group B: previously received vaccines against circovirus and porcine ileitis). The second boost given to all animals consisted of a 900 ug peptide mix corresponding to 300 ug of each viral peptide. As a control of a classical vaccination approach, two groups (7 pigs each) were vaccinated only with synthetic peptides (0.5 mg of each peptide) in combination with two different adjuvants (Group C: Montanide ISA201 and Group D: Montanide ISA206) in two different time-point doses and a boost with 300ug of the same peptide mix with the respective adjuvant and as a negative control, only PBS was injected to 5 animals (Fig. 1; Table 2).

**Table 1:** Experimental groups for exosome vaccination safety trial.

Group	Pigs	Antigen	Total ug (1 mL)	Adjuvant (1 mL)	Route
Exosomes 1 mg + M	3	Exosomes	1000	Montanide	IM
Exosomes 1 mg	3	Exosomes	1000	N/A	IM
Exosomes 0.5 mg + M	3	Exosomes	500	Montanide	IM
Exosomes 0.5 mg	3	Exosomes	500	N/A	IM
Exosomes 0.3 mg + M	3	Exosomes	300	Montanide	IM

**Table 2:** Experimental groups for immunogenicity trial.

Group	Pigs	Priming antigen	Boost antigen	Adjuvant	Route
PBS	5	PBS	PBS	N/A	IM
Group A	7	Exosomes (1 mg)/two doses	Viral peptides (300 ug each)/one dose	Montanide ISA 206vg	IM
Group B	7	Exosomes (1 mg)/two doses	Viral peptides (300 ug each)/one dose	Montanide ISA 206vg	IM
Group C	7	Viral peptides (500 ug each/two doses)	Viral peptides (300 ug each)/one dose	Montanide ISA 201 vg	IM
Group D	7	Viral peptides (500 ug each/two doses)	Viral peptides (300 ug each)/one dose	Montanide ISA 206 vg	IM

Serum samples were taken at 0, 21, 42 and 63 days post initial vaccination (dpv). Whole blood in EDTA tubes was collected at days 0 and 63. Collection of samples, maintenance and euthanasia of pigs were performed as approved by the animal use and care committee protocol of Universitat de Lleida and the Departament d’Agricultura, Ramaderia, Pesca, Alimentació I Medi rural (Section of Biodiversity and hunting) under licence DAAM 7700.

### ELISA tests

All sera samples were also blindly evaluated by a commercial ELISA (IDEXX PRRS X3 Antibody Test, IDEXX) to detect PRRSV antibodies following their own standard operation procedures (Grup de Sanejament Porcí – Lleida, Spain. http://www.gsplleida.net/cat).

Circulating IgG antibodies from vaccinated pigs in the immunogenicity trial were also evaluated by an indirect ELISA test against the synthetic peptides (ENV, N and pp1a). Plates were coated overnight at 4 °C with each peptide (5 ug/mL diluted in 50 mM Carbonate-Bicarbonate buffer, pH 9.6). Sera samples (1/100) were incubated for 1 h at room temperature, washed and incubated with secondary antibody Goat anti-Pig IgG (Fc): HRP (AbSerotec AAI41P) at 1/10000 dilution. Optical density was measured at 450 nm using Varioskan equipment (Thermo Scientific).

Antibodies were also examined using attenuated virus as coating antigen. Briefly, viral particles were diluted to reach 10^4^ particles per 50 uL with Carbonate bicarbonate buffer (pH 9.6). Plates were incubated overnight at 4 °C to ensure particle viral attachment to the plates. Plates were washed and then blocked with PBS 1×/5% non-fat dry milk. After 3 washes, 1/100 dilution of all sera were incubated at room temperature for 1 h, then washed 3X and incubated with secondary antibody Goat anti-Pig IgG (Fc): HRP (AbSerotec AAI41P) at 1/10000 dilution. Optical density was measured at 450 nm using Varioskan equipment (Thermo Scientific).

### IFN-γ ELISPOT

For evaluation of IFN-γ production, ten mL aliquots of whole blood in EDTA tubes were collected. Peripheral mononuclear cells (PBMCs) were isolated by gradient centrifugation using Ficoll Hystopaque 1077 (Sigma-Aldrich) and washed twice with PBS 1×/2% FBS after which cell density and viability were measured by trypan blue staining. Cells were resuspended in complete media “CM” (RPMI 1640, 10% FBS, 1% penicillin/Streptomycin) to have a final concentration of approximately 5 × 10^6^ cells/mL.

For ELISPOT, plates (Millipore, Cat n° MAHAS4510) were coated with 1/100 dilution of capture antibody in 50 mM carbonate/bicarbonate coating buffer pH 9.6 (Anti pig IFN-γ antibody clone P2G10 – BD Biosciences, cat n° BD-559961) and incubated overnight at 4 °C. Plates were washed twice with CM and blocked with 200uL of CM as blocking buffer for 2 hours at room temperature. Blocking buffer was discarded and all stimuli (synthetic viral peptides) were diluted in CM and added to the plate including a positive control (Lectin from *Phaseolus vulgaris* – Sigma Aldrich) and a negative control (CM alone). 100 uL of cell suspension was loaded to the plate and incubated for 48 hours at 37 °C and 5% CO_2_. Later, cells were washed out with 200uL MilliQ water and plates washed 3 times with 1X PBS/0.05% Tween 20. One hundred uL of 1/250 dilution of a biotinylated Mouse anti-pig IFN-γ clone P2C11 was loaded in the plate (BD Biosciences, Cat. n° BD-559958) and incubated 2 hours at room temperature. Detection was carried out using a 1/100 dilution of Streptavidin-HRP conjugate (BD Biosciences cat n° BD-557630) after which plates were washed four times with1X PBS/0.05% Tween 20 and incubated for 20 min with 100 uL of non-soluble substrate AEC reagent (BD Biosciences cat n° BD-551951) to reveal spots. Developing of spots was stopped by washing plates with distilled water 3 times and IFN-γ spots were counted in an AID ELISPOT Reader. Production of IFN-γ was expressed as spot forming colonies per million of PBMCs in each well.

### Statistical analysis

Statistical analysis was performed using GraphPad Prism 7. The significance level (α) was set at p < 0.05. In the ELISPOT, significant differences between groups were determined using two-way analysis of variance followed by Sidak’s multiple-comparison test. For the ELISA results, Kruskal-Wallis test for non-paired data was applied, comparing the ranks of each group in a particular timepoint and statistically significant differences were expressed as p < 0.05 (*) and p < 0.01 (**).

## Results

### Characterization of serum-derived EV-enriched fractions from non-viremic animals

**Figure 2:**
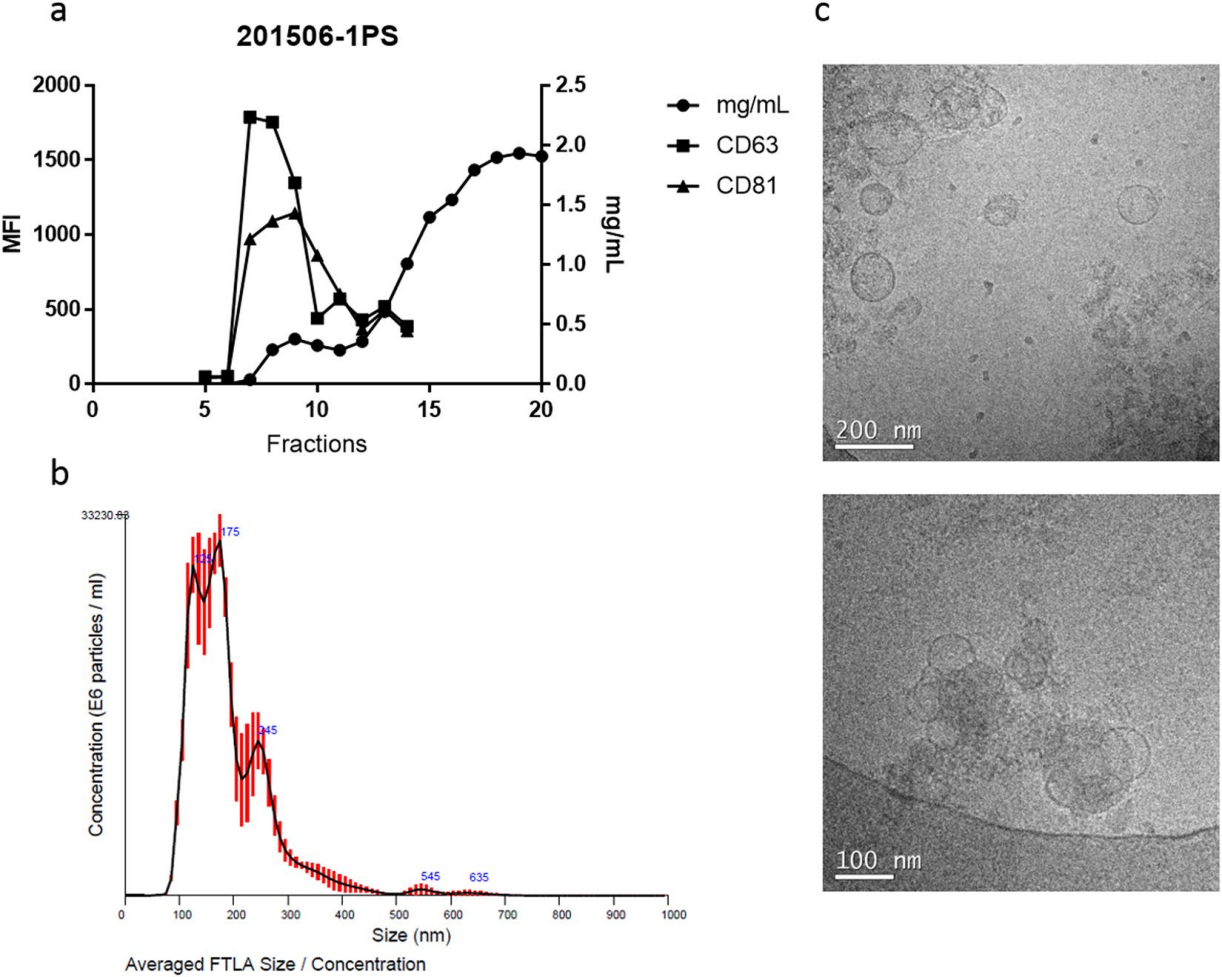
Characterisation of serum-derived enriched EV-fractions from scale-up process. (**a**) Flow cytometry analysis of CD63 and CD81. MFI, Median Fluorescence Intensity. Protein concentration by the Pierce bicinchoninic acid assay (BCA assay) is shown mg/mL. (**b**) NTA profiles of EV-enriched fractions from size exclusion chromatography (SEC). Concentration is shown in particle/ mL. (**c**) Electron microscopy. (**c**) Representative TEM images. Scales in nanometers (nm).

Size-exclusion chromatography of EV-enriched fractions eluted in fractions 7–10, were analysed by bead-based flow-cytometry to determine the presence of CD63 and CD81, two tetraspanins widely used as exosomal markers. As shown in Fig. 2a, high MFI values confirmed their presence associated with EV-enriched fractions. To further validate these results, two other tetraspanins, CD9 and CD5L, were evaluated showing similar results (Supplementary Fig. 2). Of interest, CD5L gave the highest MFI of all tetraspanins tested. As previously reported^7^, NTA analysis revealed a mean size EVs distribution of 100–200 nm (Fig. 2b) and cryo-TEM confirmed this size distribution (Fig. 2c). These data thus indicate the large heterogeneity of plasma-derived EVs. Scaled-up production of serum derived EV-enriched fractions, allowed to accurately and precisely determine protein concentration from individual SEC fractions by BCA. We calculated circa 3 mg of protein associated to EVs enriched fractions for every 180 mL of serum.

### Proteomic analysis

**Figure 3:**
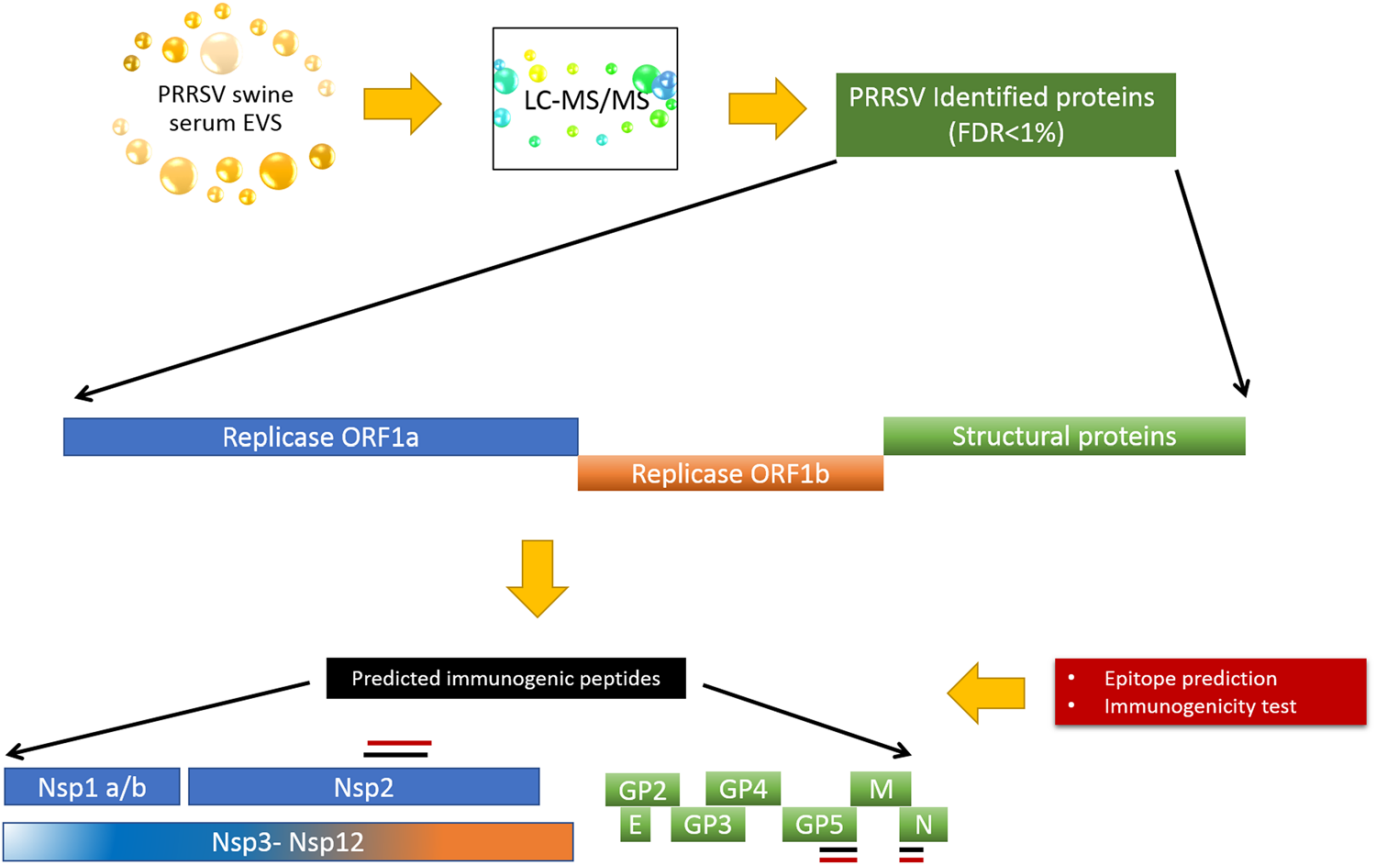
Protein identification pipeline from PRRSV convalescent sera EV-enriched fractions. Liquid chromatography (nanoLCULTRA-EKSIGENT) followed by mass spectrometry (LC-MS/MS) of EV-enriched fractions identified viral peptides with a FDR < 1% and at least two peptides from the same protein. Two different algorithms were used to examine matching regions between our identified peptides (LC-MS/MS) and possible B-cell epitopes or antigenic regions within the protein^14,15^.

Individual proteomic analysis of serum-derived EVs enriched fractions from five animals used in these vaccination trials revealed peptides corresponding to viral proteins, pp1a, Envelope glycoprotein and Nucleocapsid proteins (Fig. 3 and Supplementary Table T1). Of note, other viral proteins were detected in the preparations but were discarded due to our filtering criteria (FDR < 1% and at least two peptides from individual proteins).

After viral peptide identification, we used two different algorithms to predict their immunogenicity^14–16,19^. Interestingly, we found immunogenic regions, possible B-cell epitopes and decamers that fit as strong binders in several SLA pockets for those proteins matching with peptides detected in EVs enriched fractions. Figure 3 shows a schematic representation of the identification pipeline.

### Safety

Three different doses of EV-enriched fractions derived from PRRSV convalescent swine sera were formulated in montanide and tested in nine animals and two different doses without adjuvant were injected in six animals to assess the risk of viral presence and adverse effects of these preparations (Table 1). As a first safety control, batches were tested prior to vaccinations by a sensitive RT-PCR. None of the batches obtained individually from 180 mL of sera revealed the presence of viral particles. Moreover, none of the animals showed clinical signs associated to PRRSV, nor secondary effects due to vaccine/adjuvant preparation even after two doses of 1 mg of EV-enriched fractions. Importantly, EV-enriched fractions containing viral proteins did not induce a positive result to the gold standard ELISA test (IDEXX X3 PRRSV) suggesting that this vaccination approach could allow to differentiate vaccinated from infected animals (DIVA).

### Immunogenicity

To test the immunogenicity of serum derived EV-enriched fractions, fourteen animals were immunized twice with 1 mg of EV-enriched fractions formulated in montanide and boosted a third time with viral peptides (identified by LC-MS/MS). To compare the immunological response, two groups using a classical vaccination approach with peptides and adjuvants were used (Fig. 1). As expected from the safety data, neither clinical signs related to PRRSV infections, nor secondary effects were observed in any of these fourteen animals. Moreover, similar to the safety trial, all animals remained negative for the gold standard ELISA test for PRRSV virus, indicating that epitopes present in EVs enriched fractions and in our *in vitro* synthetic viral peptides are capable of differentiating animals infected with the virus (positive results in the ELISA test) and those vaccinated with our novel strategy.

**Figure 4:**
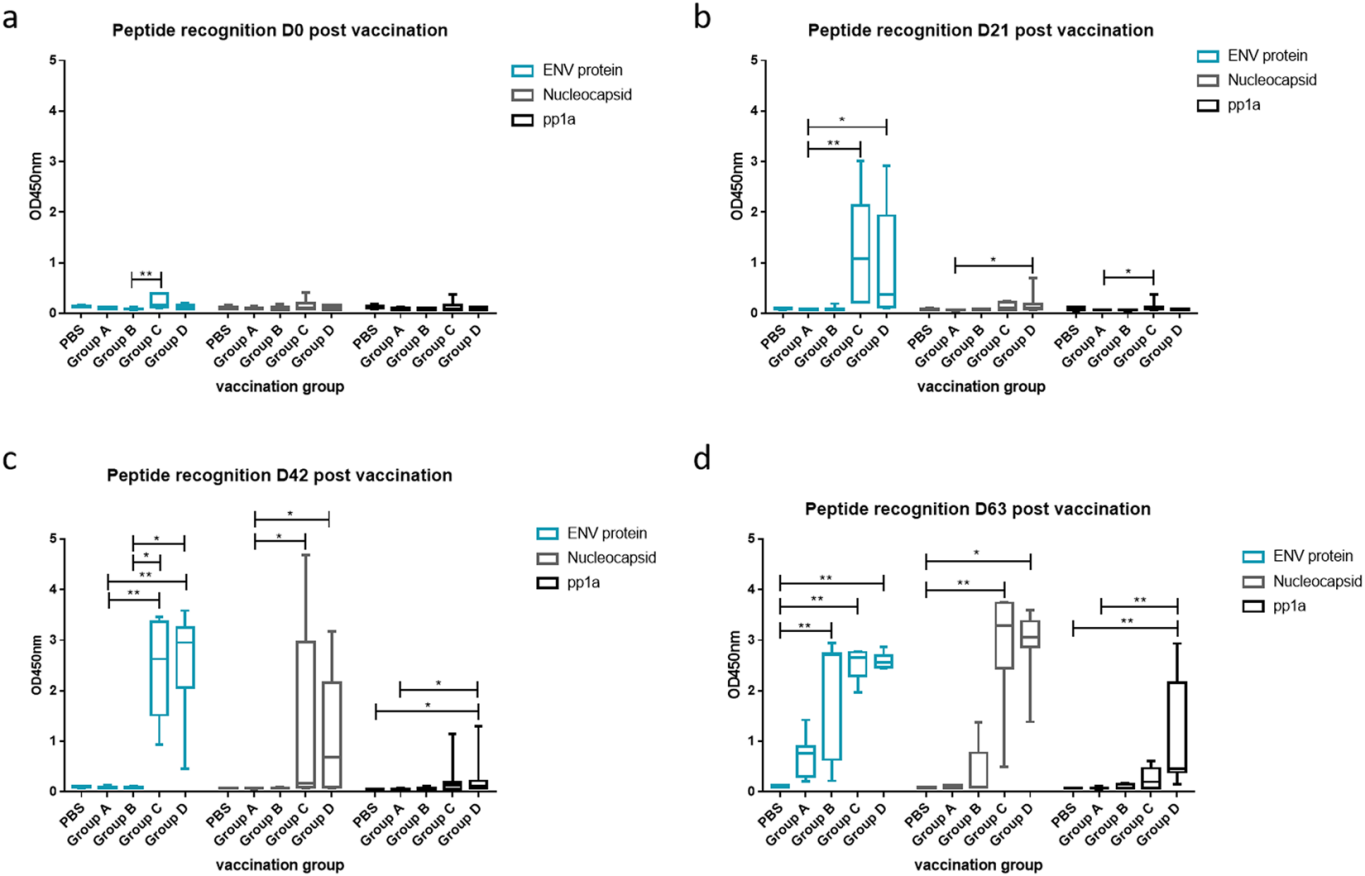
Evaluation of antibody immune response against peptides from D0 to D63 in swine sera. PRRS viral peptides were used in ELISA tests. Graphs refer to immune recognition of viral peptides after vaccination at different immunization days post vaccination. (**a**) Day cero (**b**) Day 21 (**c**) Day 42 (**d**) Day 63. Vaccination with PBS was used as a control during whole experiment. *p < 0.05, **p < 0.01. OD, optical density.

To assess humoral immune response, blood was taken from all animals at days 0, 21, 42 and 63 post-vaccination. IgG levels against the different synthetic peptides used in our immunisation approach were determined by ELISA. As expected, all animals were negative for antibodies against these viral peptides at day 0 (Fig. 4). At 21dpv, only groups C and D showed a response to the ENV peptide. At day 63dpv, higher antibody immune responses were detected in animals vaccinated and boosted with peptides as opposed to EVs-EVs-peptide ones (heterologous prime-boost) even though Groups A and B started to show a response against ENV and N but lower than Groups C and D. Moreover, based on these results, the ENV peptide seemed the most immunogenic from the ones tested, followed by the N peptide of peptide and the pp1a peptide (Fig. 4). We took advantage of the immune sera from animals that had seroconverted against the ENV and Nucleocapsid proteins to demonstrate their association with EVs used in these trials. Thus, western blot analysis of EVs used in vaccinations were performed using pre-immune and immune sera from two animals primed with EVs and boosted with peptides. These animals seroconverted after the last boost to the nucleocapsid and ENV proteins, albeit variably. Noticeably, only immune sera specifically recognized these proteins (Supplementary Fig. S3). These results thus demonstrate that viral sequences from these peptides were associated with EVs used in vaccinations. Experiments to identify residues within the 35 aa synthetic peptides representing these proteins in the context of SLA can be considered as part of a next series of targeted-pig trials including challenge.

**Figure 5:**
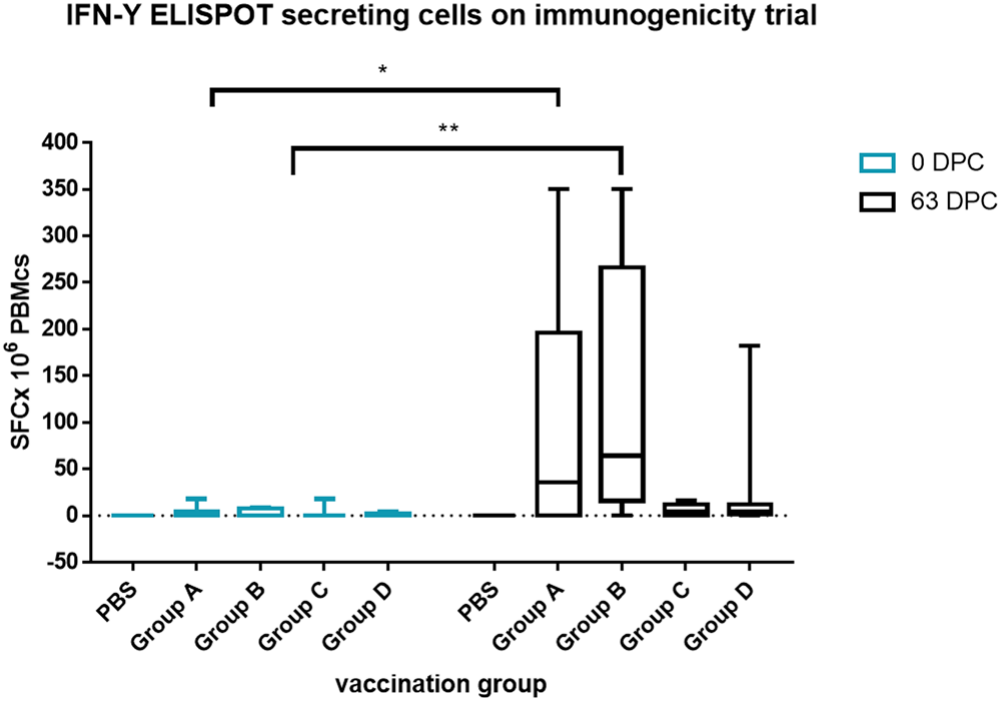
IFN-γ production after stimulation of swine PBMCs with viral peptides (mix) at days 0 and 63 post vaccination. IFN- γ producing cells were measured by ELISPOT at day cero and 63DPV. Results were compared (0dpv vs 63dpv response) using two-way ANOVA multiple comparison test. Statistically significant differences were observed only in exosome vaccinated groups between pre-vaccinated sera and 63dpv sera. *p < 0.05, **p < 0.01.

To assess cellular immunity, numbers of cells producing IFN-γ were measured by ELISPOT. Excepting for one animal in group D, remaining animals in groups C and D did not produce IFN-γ. Noticeably, there was a significant increase in number of IFN-γ producing cells with values from 60 to 350 SFCx10^6^ PBMCS on groups A and B vaccinated with EV-enriched fractions obtained from convalescence animals (Fig. 5).

## Discussion

Here, immunization of pigs with up-to two milligrams of EV-enriched fractions from sera of PRRSV convalescence animals demonstrated that they were free of virus and did not cause adverse effects. In addition, immunization of pigs with EV-enriched fractions followed by boosts with predicted immunogenic synthetic peptides from the ENV, N and pp1a proteins elicited specific humoral IgG immune responses not detected by a widely used PRRSV-diagnostics commercial test; thus, suggesting that this vaccination approach is able of differentiating infected from vaccinated animals. Lastly, such prime-boost approach elicited high and specific IFN-γ responses in comparison with immunizations using peptides alone.

Previous studies identified viral peptides corresponding to the Nucleocapsid, and pp1a proteins in serum-derived EV-enriched fractions from pigs that had overcome PRRSV infections^7^. Here, we confirm such results as we identified viral peptides from these same two proteins, in addition to peptides from envelope GP5 protein, in individual mass spectrometry studies of five different convalescence animals. Our data strongly indicate that there is selective viral antigen-cargo associated with EV-enriched fractions from convalescence animals. The Nucleocapsid protein is an important structural protein which after cytoplasmic synthesis migrates to the nucleus suppressing expression of type I interferons^20,21^. Moreover, interactions between this structural protein and antigen presenting cells in the presence of viral induced IL-10 can lead to and alteration of APC functions^21^. pp1a encodes 10 non-structural proteins, NSPs, and expression of PRRSV nsp1α/β and nsp2 were found to exert strong inhibitory effects on IFN-β promoter activation^22^. In addition, it has been recently discovered that the pp1a region of highly pathogenic PRRSV strains plays an important role in inducing neutralizing antibodies in piglets^23^. The GP5 protein contains epitopes involved in antibody dependent virus neutralization, protection, cell recognition and binding^24^. However, it remains to be studied which EV component(s) are responsible for the immunogenicity presented in this work. EVs include sets of immune related proteins such as MHC that could contribute to elicit a better immune response^25^. Recently, we have analyzed the peptides present in EV by NetMHCSpan and our preliminary results identified peptide sequences with the theoretical ability to bind to certain MHC (SLA in the case of swine). Moreover, these peptides were predicted as strong binders for different SLA alleles (data not shown). If these predictions are confirmed, such antigenic peptides in the EV cargo could participate in developing specific immune responses.

Our data thus strongly suggest that novel viral antigenic peptides can be discovered in serum EV-enriched fractions from animals that were free of virus in peripheral circulation. As it is known that PRRSV infection can last for months in internal tissues^3,26,27^, it is tempting to speculate that such cryptic infections in lymphoid tissues might release into circulation EV-enriched fractions with selected cargo eliciting immune responses capable of maintaining animals with undetected viremias in peripheral circulation and with no clinical symptoms.

To scale-up the production of EV-enriched fractions for the safety and immunogenicity trials, serum samples were concentrated 100-times through ultracentrifugation and size exclusion chromatography (see Methods). To demonstrate that these preparations were free of virus, a sensitive qRT-PCR capable of detecting 100 particles/mL (Applied Biosystems) was firstly performed showing that they did not contain viral RNA. Next, we injected increasing doses of EV-enriched fractions into nine different animals in safety trial and fourteen animals in the immunogenicity trial (Fig. 1). Results demonstrated that injections of up to two milligrams of serum-derived EV-enriched fractions from these animals did not cause any clinical symptoms and were free of virus (Tables 1 and 2). Of interest, a recent publication suggested that exosomes from PRRSV-infected cells and free of virus act as vehicles for intercellular transmission^28^. Whether these seemingly discrepant results are due to *in vitro* vs *in vivo* studies, remains to be determined.

Current vaccine approaches against PRRSV have met little success in developing broad neutralizing antibodies and no correlate of protection is presently available with antibody levels^29^. Moreover, available tests for measuring neutralizing antibodies are difficult to interpret as the PRRSV isolate used for testing are not field strains but cell-cultured adapted^30^. Our data demonstrated that our prime-boost approach elicited specific IgG antibodies. However, even though all animals seroconverted at the end of the study, titers and recognition against individual peptides varied significantly among groups (Fig. 3). Whether these antibodies contain high quality neutralizing antibodies remains to be determined. Serum-derived EV-enriched fractions represent a complex mixture of vesicles release from widely different type of cells^31^. Therefore, it is reasonable to speculate that serum EVs containing viral peptides represent a very small percentage of circulating EVs, partly explaining the low seroconvertion rate in EV-vaccinated groups when compared with peptide vaccination added to individual variability observed in this trial.

Highly pathogenic PRRSV strains are emerging all over the world with cases mainly in Asia, America and some in Europe through recombination of wild type viruses with vaccine strains^30,31^ making a top priority to find a test that allow identification of vaccinated animals from those that overcome the disease (DIVA). As several current vaccination approaches are using modified live viruses, deletion mutants and chimeric viruses are being tested with the idea of solving the above mentioned problem^32^. Noticeably, based on a gold standard commercial method (IDEXX PRRSV x3 Ab test), sera from all animals that seroconverted were diagnosed free of virus suggesting that this vaccination approach can differentiate infected from vaccinated animals.

Priming with EV-enriched fractions and boosting with viral peptides induced a high and specific cellular IFN-γ immune response above 300 SFCx10^6^ PBMCs in EVs vaccinated groups, that was not present in the peptide-vaccinated group. The fact that viral peptides alone were not able to stimulate immune cells *in vivo* and EV-peptide vaccination approach could, made this a new and exciting opportunity to evaluate the role of EV during and post natural infection, as well as their role in viral clearance and immunity. Our result was remarkable when considering that in natural PRRSV infections, T cell immune responses are usually weak and delayed^33^, as well as variable appearing 4–6 weeks after infection^34^. Moreover, infected pigs show an important decrease in CD8+ T-cells and IFN-γ production and a reduction of 50 to 80% of NK cell cytotoxicity from day 7 to 24 post infection maybe due to increased IL-4 levels^35^.

Interestingly, exosomes are vehicles of proteins and nucleic acids acting in intercellular communication and antigen presentation^36–39^. Thus, serum-derived EVs from convalescence sera, bearing viral proteins and other immune-related proteins could be delivered to sites where memory cells are present in higher frequencies and activate effector memory T cells. In the absence of supporting data, this hypothesis remains to be proved. Such mechanism, however, has been recently shown to be elicited by reticulocyte-derived exosomes from infections in an experimental rodent malaria model^40^.

Because of their ability to modulate the immune response, exosomes are presently being explored as novel therapeutic agents against infectious diseases. Pioneering studies demonstrated that macrophages infected with *Mycobacterium bovis* secreted exosomes inducing bacterial-specific pro-inflammatory activity^41^. Similar results were obtained with exosomes from macrophages infected with *Mycobacteium tuberculosis* and this response was also evident in other infectious diseases caused by intracellular pathogens, *Salmonella typhimurium*, *Toxoplasma gondii, Plasmodium yoelii* as well as in other parasitic diseases from worms and protozoa^41,42^. Remarkably, using serum-derived CD80+ and CD80- enriched exosomes from *Eimeria* infected chicken induced increased numbers of intestinal and spleen cells secreting Th1 (IL-2, IL-6, IFN-γ) and Th2 (IL-4) cytokines, compared with unimmunized controls. In addition, in the case of *Eimeria*, CD80+ EVs induced a stronger immune response with increased numbers of IFN-γ and IL-2 producing cells (in gut and spleen as well) and greater protective immunity following *E. tenella* challenge, as measured by weight gain, feed efficiency, parasite shedding, and intestinal lesions^9^. Altogether, the data from several studies strongly support the use of EVs and exosomes as a novel vaccine approach against veterinary diseases of economic importance.

## Electronic supplementary material

Supplementary information

Supplementary Table I
