# Supplementary material for: Targeted-pig trial on safety and immunogenicity of serum-derived extracellular vesicles enriched fractions obtained from Porcine Respiratory and Reproductive virus infections
Source: Sci Rep. 2018 Nov 30;8:17487. doi: 10.1038/s41598-018-36141-5 (PMC6269534; doi:10.1038/s41598-018-36141-5)

Targeted-pig trial on safety and immunogenicity of serum-derived extracellular vesicle enriched fractions obtained from Porcine Respiratory and Reproductive virus infections

Running title: Safety and immunogenicity of serum-derived PRRSV extracellular vesicles

Sergio Montaner-Tarbes[1,2], Elena Novell[3], Vicens Tarancón[3], Francesc E. Borrás[1,4], Maria Montoya[1,5], Lorenzo Fraile[1,2*] and Hernando A del Portillo[1,4,6,7*]

[1]Innovex Therapeutics S.L, Badalona, Spain

[2]Departamento de Ciència Animal, ETSEA, Avenida Alcalde Rovira Roure, 191, Universidad de Lleida, Lleida, Spain

[3]Grup de Sanejament Porci, Lleida, Spain

[4]Germans Trias i Pujol Health Science Research Institute (IGTP), Can Ruti Campus, 08916 Badalona, Spain

[5]Centro de Investigaciones Biológicas, CSIC, Madrid, Spain.

[6]ISGlobal, Hospital Clínic - Universitat de Barcelona, Barcelona, Spain. C/ Roselló 153, 08036 Barcelona, Spain

[7]Institució Catalana de Recerca i Estudis Avançats (ICREA). Passeig Luis Companys 23, 08010 Barcelona, Spain

*Equal correspondence to:

hernandoa.delportillo@isglobal.org / Lorenzo.fraile@ca.udl.cat

Key words: extracellular vesicles, vaccines, targeted-pig trial, safety, immunogenicity, Porcine Reproductive and Respiratory Syndrome Virus, PRRSV

- **Supplementary figures.**

    - **Supplementary figure S1**. Individual FACS and protein elution profile of all prepared batches of isolated exosomes from convalescent swine sera for PRRSV.

    - **Supplementary figure S2.** FACS and protein elution profile for evaluation of tetraspanins from serum EVs enriched fractions (CD5L, CD9, CD63 and CD81). **(a, c)** Serum sample 201506-1PS (**b, d)** Serum sample 201506-6PS.

    - **Supplementary figure S3.** ***Western blot analyses of EVs used for vaccination.*** **(A) ELISA.** PRRS viral peptides (GP5, Nucleocapsid and ORF1a) were used in ELISA tests. Graphs refer to immune recognition from individual pigs primed with EVs and boosted with peptides at day 63 post-vaccination. OD, optical density. **(B) WESTERN BLOT**. Twenty micrograms of *EVs* from the same batches used in vaccinations were resolved on 12% SDS-PAGE and transferred to nitrocellulose membranes. Nitrocellulose membrane was cut and individual strips were incubated for 1h with preimmune and immune sera at 1:100 dilution. Secondary antibodies were used at 1:5000 and 1:10000 dilutions. All strips were evaluated together during developing process at the same time and exposure. Signals were detected on a chemioluminescence BOX Syngene device using standard measurement of exposure and 1 photo per minute are shown below since minute one. GP5 (*) and Nucleocapsid protein (**). Molecular weight in kiloDaltons (kDa).

- **<u>Supplementary tables.</u>**

    - **Supplementary table T1**. Proteins identified by Mass spectrometry and Maxquant software.

- **Supplementary figure S1.**

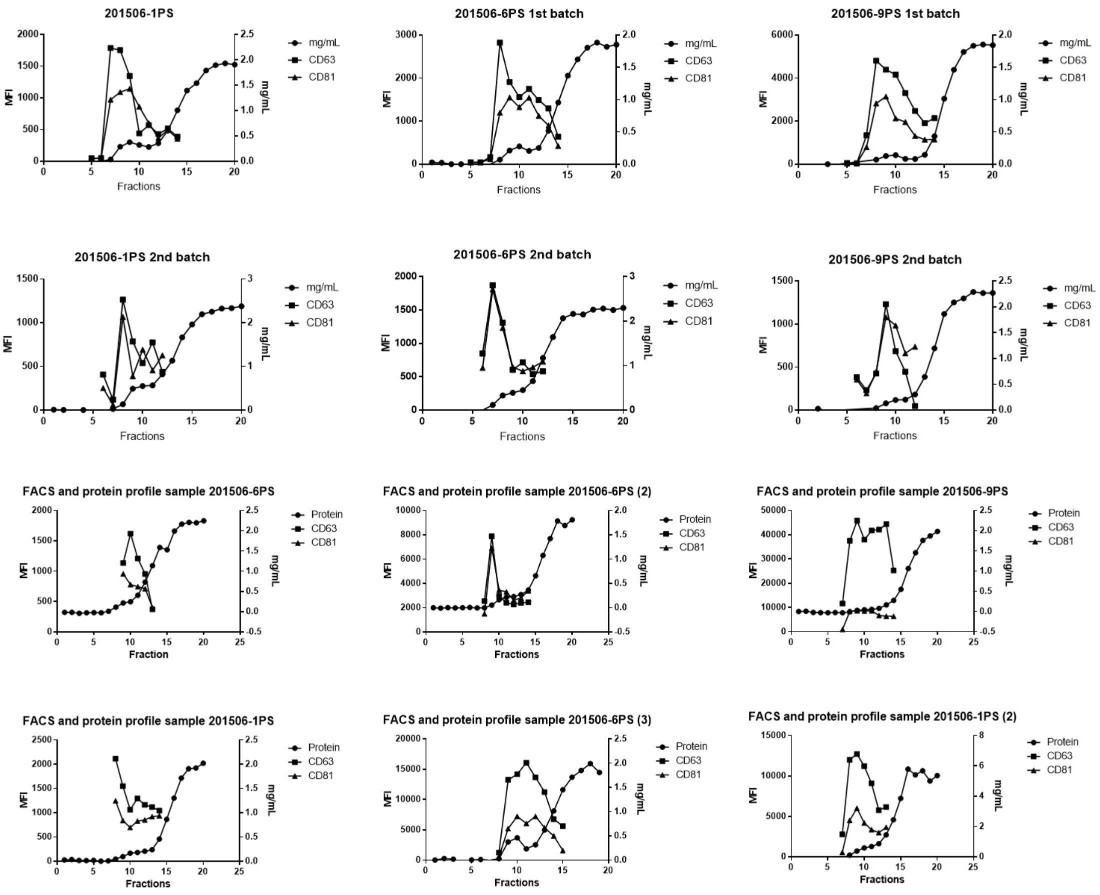

- **Supplementary figure S2.**

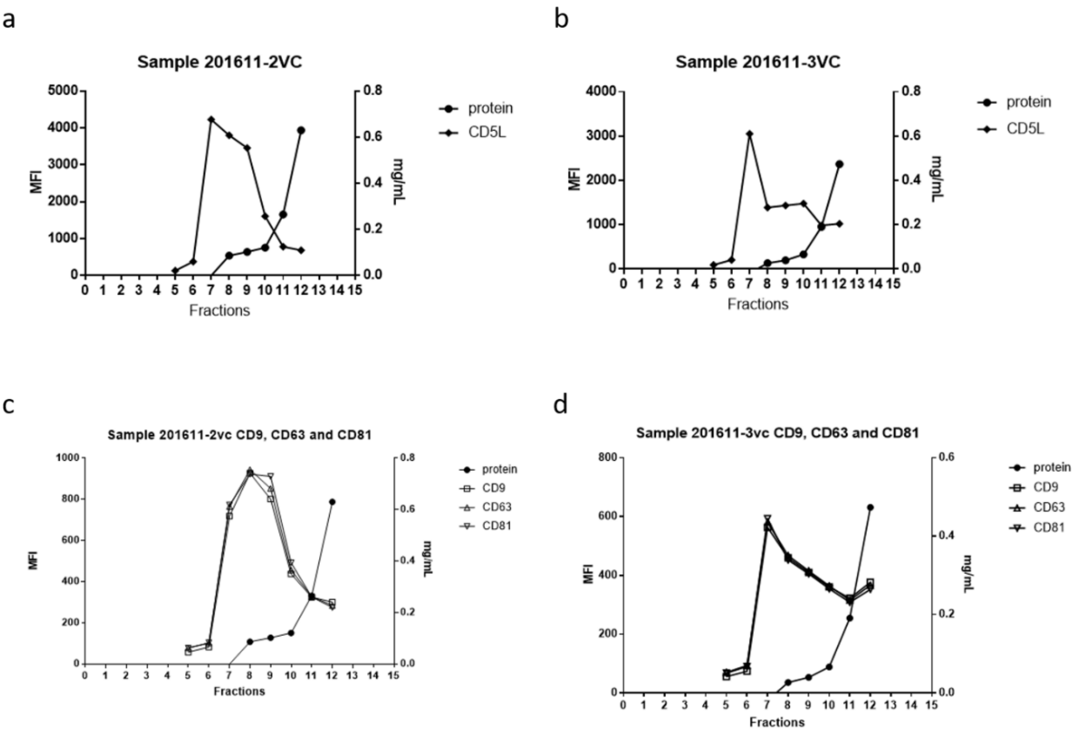

- **Supplementary figure S3**

A

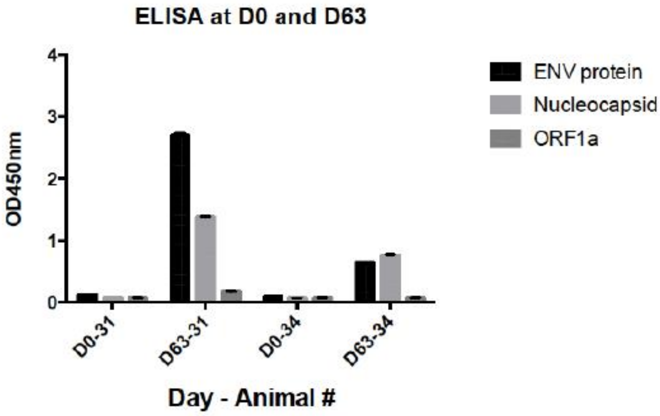

B

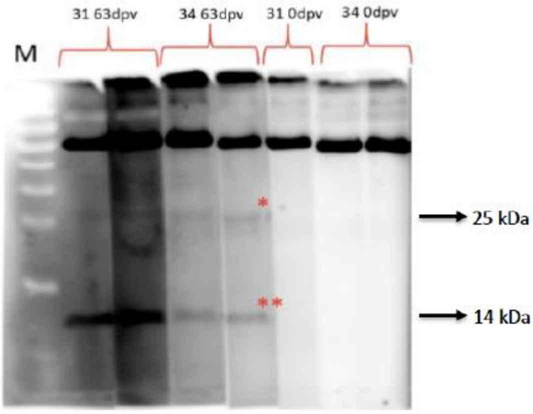

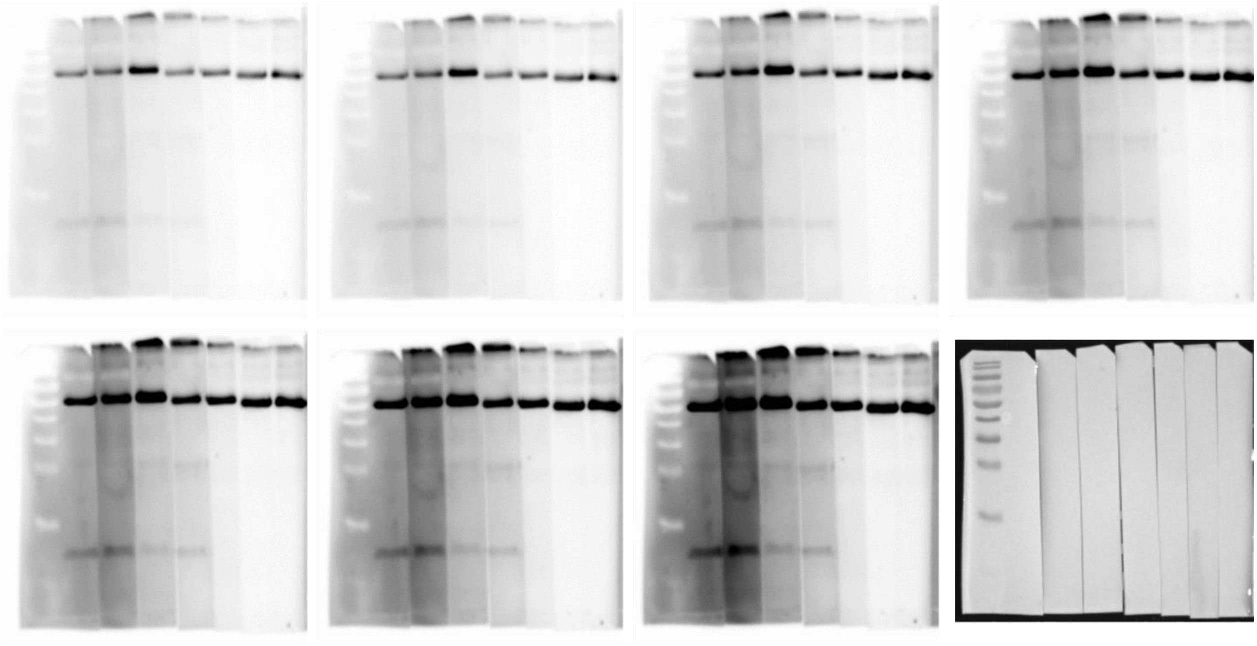

Supplement: Supplementary file 1 — Supplementary information [file 41598_2018_36141_MOESM1_ESM.pdf]
